# West African Trypanosomiasis with Central Nervous System Involvement

**DOI:** 10.4269/ajtmh.16-0117

**Published:** 2016-09-07

**Authors:** Vicente Abril, José Luis Ramos

**Affiliations:** ^1^ Department of Infectious Diseases, Consorcio Hospital General Universitario, Valencia, Spain; ^2^ Department of Microbiology, Consorcio Hospital General Universitario, Valencia, Spain

**Figure 1.**
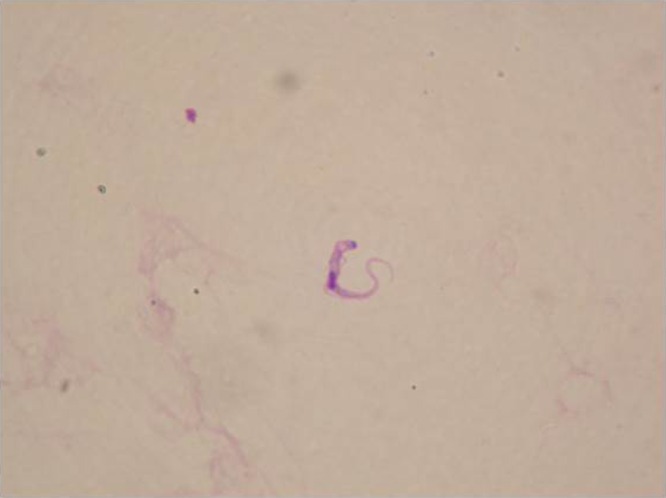
*Trypanosoma brucei* trypomastigote form in CSF. Giemsa stain, magnification ×100.

We report the case of a 17-year-old man from Mbimi, Equatorial Guinea, who presented with several months' history of episodic fever and generalized lymphadenopathy. Physical examination showed 2–3 cm neck, axillary, inguinal, submaxillary, and epitrochlear, bilaterally symmetrical rubbery lymph nodes. Erythrocyte sedimentation rate was 120 mm/hour. A full-body computed tomography scan confirmed the presence of lymphadenopathies as well as homogeneous hepatosplenomegaly. Excisional lymph node biopsies showed a reactive lymphadenitis with follicular hyperplasia. Serology of sleeping sickness (*Trypanosoma brucei*) performed by indirect immunofluorescent assay IgG and plasma detection of parasite nucleic acid by polymerase chain reaction (PCR) were both positive. Lumbar puncture was performed, obtaining a cerebrospinal fluid (CSF) with 10 white blood cells (WBCs)/μL showing a few trypomastigotes of *T. brucei* (Figure 1

). *Trypanosoma brucei gambiense* PCR in CSF and lymph node tissue were also positive. The patient completed treatment with eflornithine for 14 days and total resolution of symptoms was achieved. Three months later, a second PCR in CSF was negative and WBCs were 3/μL.

Examination of CSF plays an essential role in diagnosis, selection of treatment, and patient follow-up in African trypanosomiasis.^1^ A lumbar puncture should be performed at diagnosis for stage determination and at the end of treatment to assess cure. The presence of trypanosomes defines second-stage disease.^2^ Detection of parasite nucleic acids by PCR is useful as a more sensitive approach.^3^ The number of WBC in CSF is important in both diagnostic and follow-up test. Thresholds of ≤ 5 WBC/μL are considered cure and ≥ 50 should be considered relapse.^4^ Even though it is recommended to reassess response to treatment at 6 months, in this particular case, the lumbar puncture could not be repeated because of the patient's lack of consent. We used eflornithine alone with good results, but now combination with nifurtimox is considered the standard of care by the World Health Organization.^5^
